# Supplementary material for: Are carbon emissions trading and green financial instruments synergistic? -Comprehensive quantitative research based on content analysis
Source: PLoS One. 2024 Mar 7;19(3):e0298601. doi: 10.1371/journal.pone.0298601 (PMC10919601; doi:10.1371/journal.pone.0298601)
Supplement: S1 Data — (ZIP) [file pone.0298601.s001.zip › Data/地级市Z_F工作报告.zip!/╣π╬≈/│τ╫≤╩╨/└┤╫╘╣1⁄2╓┌║┼í╛┬φ┐╦╩2╛▌═°í┐_2014.docx]

各位代表：

　　现在，我代表市人民政府向大会作政府工作报告，请予审议。同时，请市政协委员和其他列席会议的同志提出意见。

　　一、 2013 年工作回顾

　　 2013 年，是全面落实党的十八大精神的开局之年，是加快 “ 十二五 ” 规划建设的重要一年。一年来，面对错综复杂的形势，全市各族人民在自治区党委、政府和市委的正确领导下，坚持 “ 稳中求进 ” 的总基调，以 “ 加力开放合作、加快南崇经济带建设 ” 为主题，在 “ 稳增长、调结构、促转型 ” 上狠下功夫，迎难而上，真抓实干，全力推进 “ 四大建设 ” 、 “ 五个打造 ” ，保持了经济社会持续健康发展，开创了 “ 把崇左建设成为面向东盟开放合作的新高地、成为提升广西沿边开放水平的桥头堡 ” 的新局面。

　　 —— 经济运行稳中有进。初步统计，全年生产总值完成 584.63 亿元，比上年增长 10.2% ，连续 10 年实现两位数增长，增速与全区持平、高于全国 2.5 个百分点；财政收入完成 73.02 亿元，增长 10.6% ，总量保持全区第 9 位；固定资产投资完成 450.84 亿元，增长 24.7% ，增速高于全区 2.9 个百分点。全市经济实现 “ 总体平稳、稳中有进 ” 。

　　 —— 产业发展提质增效。农业生产稳步增长，第一产业增加值增长 4.2% 。工业结构优化升级，工业经济效益综合指数同比提高 60 个百分点，规模以上万元工业增加值能耗下降 4.4% 。现代服务业加快发展，第三产业增加值增长 7.8% 。结构调整和产业升级呈现积极变化，三次产业结构调整优化为 26 ∶ 42 ∶ 32 。

　　 —— 工业经济后劲增强。全市规模以上工业总产值突破 500 亿元大关，达到 523.7 亿元；规模以上工业增加值完成 204.2 亿元，增长 17% ；工业对经济增长的贡献率达到 55.9% ，提高 5.6 个百分点。工业经济实力持续提升，对经济增长的拉动日趋增强。

　　 —— 外贸水平全区领先。外贸进出口总额达到 102.8 亿美元，成为广西第一个突破 100 亿美元的地级市，占全区总额的 31.3% ，比上年增加 31.5 亿美元，占全区增量 90% 以上。外贸进出口总额连续 5 年稳居全区第一，出口总额连续 7 年稳居全区第一。

　　 —— 民计民生持续改善。人民生活水平进一步提高，城镇居民可支配收入 21289 元，增长 9.9% ，增速排全区第 6 位；农民人均纯收入 7077 元，增长 13% ，比全区人均水平高 287 元；贫困人口减少 6 万人。城镇登记失业率 2.02% ，低于自治区控制数 2.18 个百分点。城乡消费市场更加活跃，社会消费品零售总额 96.38 亿元，增长 14.2% 。

　　 —— 重点工作取得突破性进展。我们立足当前、着眼长远、凝心聚力，努力推进一批事关崇左长远发展的重大项目和重点工作，取得了突破性进展。主要有十件大事：一是抢抓中越两国签署《关于建设中越跨境经济合作区的谅解备忘录》重大机遇，积极推进中越凭祥 — 同登跨境经济合作区申报和建设，自治区政府已行文上报国务院，有望今年 6 月获得中越两国政府批复实施。二是积极争取设立广西凭祥重点开发开放试验区，着力推进南宁 — 崇左 — 凭祥对外开放经济带建设，已获列入《国务院关于加快沿边地区开发开放的若干意见》。三是崇左市整体列入北部湾经济区政策实施范围，对于我市推动重大项目和产业园区建设、促进开放合作、优化投资环境等方面将起到极大的促进作用。四是积极融入 “ 双核驱动 ” 战略格局，推动崇左市整体纳入珠江 — 西江经济带，给崇左发展带来新机遇。五是成功争取将崇左市整体纳入国家发改委编制的《左右江革命老区振兴规划》，为崇左市加快发展助添新动力。六是中泰 ( 崇左 ) 产业园建设取得重大进展，得到自治区党委、政府主要领导和泰国政府的支持，在泰国政商界形成了合作建设的广泛共识。七是南国铜业项目环评获得环保部正式批复，中铝稀土国盛稀土分离第一条生产线将在今年上半年投料试产。一批产业项目相继开工或竣工投产，新的经济增长点和支柱产业正在形成。八是友谊大道北段延长线、环城东路改扩建工程、崇左国际客运中心等一批市政项目竣工投入使用，中心城区基础设施进一步完善。九是精心筹备、积极推进园博会申办工作，获得第七届广西园林园艺博览会承办权。十是开工建设城南小学，整合五所中职学校组建广西崇左东盟国际职业教育学院，启动建设市体育中心项目，城市公共服务设施日趋完善。一年来，我们主要做了以下工作：

　　 ( 一 ) 着力推进 “ 两加 ” 工作，开放合作水平明显提升。全面拓展开放合作深度与广度。进一步深化区域交流合作，积极推动南北钦防玉崇百区域经济一体化发展，共同加强旅游、物流、交通合作；主动参与北部湾经济区开放开发和西江经济带、桂西资源富集区建设。加快与东盟互联互通建设，崇左至靖西高速公路建设全线铺开，崇左至水口、隆安至硕龙高速公路等项目前期工作加快推进；中越两国正式签订水口 — 驮隆中越界河公路二桥项目建设协议书；中越公务及货运直达车辆实现跨境通行；爱店、硕龙、水口等口岸升格和扩大开放工作取得新进展。积极开展国际友城工作，与越南谅山市建立国际友城关系，与缅甸勃固市、柬埔寨腊达那基里省、波兰比亚韦斯托克市签署友城意向书；各县 ( 市、区 ) 友城工作取得新突破。大力实施南崇经济带发展规划，加大园区建设投入，年内完成基础设施投资 14.27 亿元，增长 85.6% ，园区基础设施明显改善，承载能力进一步增强。大力推进特色园区建设，广西 · 东盟特色食品产业园产业发展布局规划通过评审，广西糖果休闲食品产业园完成控制性详细规划编制。全年园区工业总产值完成 368.91 亿元，增长 29.4% 。

　　 ( 二 ) 着力推进重大项目建设，经济发展后劲不断增强。突出抓好产业、交通、城建等重大项目建设，落实市领导联系推进重大项目责任制，促进了一批重大项目开竣工。全市在建 5000 万元以上重大项目 441 个，年内完成投资 271.15 亿元，重大项目占投资总额的 60.1% ，拉动作用明显。红狮水泥等项目开工建设，宁明丰浩甘蔗循环经济、南方水泥、龙州东盟国际商贸城等项目进入全面施工阶段，中信大锰天等公司电解锰、扶绥金大电动车等项目实现竣工投产。大力推进招商引资，中粮、中铝集团等央企，科创、雅客、南方铜业等名企纷纷入崇，势头向好，粤港澳招商成果显著，全年市外境内招商引资项目到位资金 383 亿元，增长 24.8% ；全口径利用外资项目到位资金 2.44 亿美元，增长 60.5% 。

　　 ( 三 ) 着力优化产业结构，新型工业化进程持续加快。推动支柱特色产业规模不断扩大、档次不断提升。糖业方面，大力发展糖深加工和糖业循环经济，拉长产业链，提高产品附加值，全年糖业循环产业产值 264.2 亿元，增长 15.7% 。矿业方面，通过丰水期电价政策的激励和市、县两级价格调节基金的带动，锰企业进一步恢复和扩大生产，锰业开工率从去年 5 月份的 46.4% 提高到 71.4% 。全年锰业产值 96.47 亿元，增长 18.1% 。同时，铜、稀土、铝等新产业不断发展，项目建成后将进一步丰富我市产业体系。红木产业方面，红木产业加工基地一期配套基础设施建设基本完工，首批入园企业已有 2 个项目实现竣工投产。 “ 白黑红 ” 产业规模以上工业总产值占全市比重达到 70.4% 。强优企业不断发展壮大，全市规模以上企业达 150 家，比去年增加 14 家；全市共有 11 家企业上榜 2013 年全区纳税百强企业，户数排名全区第二。非公经济快速发展，全年实现地区生产总值 305.14 亿元，占全市比重的 52.1% ；非公工业对工业增长的贡献率达到 75.6% 。

　　 ( 四 ) 着力发展现代农业，农业农村经济持续发展。实施农民收入倍增计划，现代农业加快发展。粮食生产综合能力稳步提高，粮食总产量达 50.79 万吨，增长 4.2% 。积极引导推动农村土地流转，累计流转面积达 79.6 万亩，完成 “ 小块并大块 ” 土地整治 42 万亩。实施甘蔗高效节水灌溉工程，完成 18.43 万亩。农业产业化水平不断提高，新增农民专业合作社 137 家，累计达 452 家。大力发展特色养殖和林下经济。加快水利建设，累计完成冬春水利建设投资 10.55 亿元，实现新增恢复和改善灌溉面积共 38.96 万亩。大力推进农业机械化，获农机购置补贴资金总量排全区第一，建立甘蔗生产全程机械化示范片 42 片，农业耕种收综合机械化水平达 44.5% 。

　　 ( 五 ) 着力推进财税金融建设，经济支撑能力持续增强。面对糖、锰价格持续低迷等不利因素的影响，广开财源抓增收，优化结构抓支出，财政运行呈现增长平稳、支出有序的态势。严格控制非税收入增长。支出结构进一步优化，全年公共财政预算支出累计完成 140.92 亿元，增长 7.6% 。金融信贷运行平稳，各金融机构本外币各项存贷款余额持续增长，贷款余额增速排名全区第 1 位。金融服务能力明显提升，新增融资性担保公司 1 家和小额贷款公司 3 家。积极开展政银企合作，与建行广西分行、国家开发银行广西分行分别签订金融合作协议，其中与国开行的协议贷款金额达 100.3 亿元，第一批贷款 13.5 亿元已落实到位。

　　 ( 六 ) 着力建设美丽崇左，城乡面貌持续改观。以 “ 创业十年 · 美丽崇左 ” 活动为契机，大力推进城市建设三年大会战和建市十周年献礼工程。市委党校新校区、市文化艺术中心、八大住宅区等一批重大项目相继竣工，水口湖整治、天然气管道等工程实现开工。教育、文化、卫生、市场、路网等城市配套功能不断完善，框架不断拉大，城市形象不断提升。加快连接中心城区与崇左城市工业区的路网建设，工业大道建成通车，有力推动了 “ 产城互动、园城一体 ” 发展。大力实施 “ 书记工程 ” 小城镇建设、城乡风貌改造、农村危旧房改造，全面开展 “ 美丽广西 · 清洁乡村 ” 活动，城乡人居环境进一步改善，城镇化发展质量有效提升，全市城镇化率预计达 34.4% ，提高 1.5 个百分点。积极推进生态市创建工作，全面开展环境安全隐患整治行动，年度节能减排目标任务全面完成，全市环境质量总体保持良好。 “ 绿满八桂 ” 造林绿化工程综合排名全区第一，森林覆盖率提高到 54.21% 。生物多样性保护取得重大突破，荣获 “ 中国白头叶猴之乡 ” 称号，大新恩城自然保护区获批成为我市第三个国家级自然保护区。

　　 ( 七 ) 着力发展文化旅游业，旅游品牌创建初见成效。实施 “ 快乐崇左 ” 工程，提升 “ 吃、住、行、游、购、娱 ” 档次。加快文化与旅游融合发展，花山岩画文化景观申遗工作加快推进。《左江流域旅游发展总体规划》已通过专家评审。大力实施旅游 “25671” 工程。扶绥龙谷湾、龙州 “ 发现 · 弄岗 ” 等项目实现开工，中国 — 东盟旅游集散中心等项目建设扎实推进，崇左国际大酒店、天湖大酒店、凭祥电影城等建成投入使用。大新县、凭祥市被列入首批广西特色旅游名县创建名单。德天瀑布景区创建 5A 工作加快推进，免税购物区申请工作有序开展。全年接待游客总人数 1141.28 万人次，增长 15.8% ；旅游总收入 80.06 亿元，增长 19.7% 。

　　 ( 八 ) 着力保障和改善民生，社会持续和谐稳定。全市财政用于民生领域支出 95.4 亿元，占公共财政预算支出 67.7% 。 10 项为民办实事工程全面完成，累计完成投资 36.93 亿元。超额完成保障性安居工程年度目标任务，累计投资 6.73 亿元。滇桂黔石漠化片区区域发展与扶贫攻坚扎实推进，兴边富民行动大会战完成投资 5.1 亿元。教育事业协调发展，教育惠民工程全面落实，九年义务教育巩固率、高中阶段教育毛入学率分别提高到 74.5% 、 70.9% 。成功举办崇左市第二届运动会暨第三届少数民族传统体育运动会。科技创新和推广成效突出，每万人口发明专利拥有量增长 200% ，增幅排名全区第一。大力推进食品药品监管体制改革，深入开展食品药品专项整治，保障食品药品安全。卫生工作有序推进，实施基本公共卫生服务项目，推进医药卫生体制改革，巩固完善国家基本药物制度；与广西医科大学签订战略合作框架协议，协作共建广西民族医院。社会保险覆盖面进一步扩大，基本社会保险覆盖率达到 85% 。民政事业健康发展，低保、五保供养保障水平不断提高，中国乐养城被列入自治区社会化养老服务试点。全面推进诚信计生，人口自然增长率稳定在责任指标内，出生人口性别比持续下降。物价总水平基本稳定，全市 CPI 上涨 2.5% ，控制在 4% 的年度调控目标内。深入开展民族团结进步创建活动，民族团结进步事业得到巩固和发展。国防教育和后备力量建设深化开展，双拥共建工作有序推进。全面彻底开展安全生产大检查，安全生产形势持续稳定好转。继续加强和创新社会治理，深化平安广西建设工作会议在我市召开，平安建设工作走在全区前列。在敏感节点、重大活动期间实现 “ 六个坚决防止 ” 目标，全市公众安全感和满意度继续保持全区第一。政法综治维稳工作成为全市一张靓丽的名片和品牌 !

　　 ( 九 ) 着力加强政府自身建设，行政效能持续提升。积极开展党的群众路线教育实践活动。严格贯彻落实 “ 八项规定 ” ，积极深入基层调查研究和解决实际问题。主动接受人大监督和政协民主监督，定期向市人大、市政协班子通报情况，听取意见建议。创新开展问政工程，在广西首次举办电视问政直播活动，自觉接受群众监督，积极回应群众关切。全年共办理人大代表建议 56 件、政协提案 103 件。全面推进简政放权工作，最大限度地下放行政管理权限，接收自治区下放行政审批事项 112 项，市本级下放行政审批事项 17 项，取消行政审批事项 76 项。加强各级政务服务中心建设，市政务服务中心审批部门进驻率、审批事项进入率、群众满意率均达 100% ，优化办事流程，推行一体化服务，行政效能和服务水平不断提升。结合市情创新开展体制机制改革，进一步理顺市与江州区权责关系，解决市、区财政体制存在的突出问题，推进城市工业区体制改革、投融资平台建设。廉政建设扎实推进，行政监察和审计监督更加规范，政风、行风建设进一步加强。

　　一年来，我市人力资源、机构编制、统计、国有资产监督管理、法制、水库移民、供销合作、二轻、工商行政管理、质量技术监督、新闻出版、广播电视、宗教、侨务、涉台事务、机关事务管理、驻邕管理、无线电管理、气象、水文、消防、边防、海关、海事、出入境检验检疫、档案、信访、保密、国家安全、调处、老龄、红十字会、地方志等工作整体推进。供电、通信、邮政、石油、烟草、盐业等行业和工会、共青团、妇联、科协、文联、侨联、残联、工商联、社科联等群团组织在经济社会发展中发挥了重要作用，作出了积极的贡献。

　　各位代表，过去的一年，异常复杂的经济形势，对崇左是一场严峻考验，对各级干部是一场综合测试。我们重实干、出实招、求实效，推动崇左经济社会向前迈出了艰辛而又坚实的一步。一年来，市委坚强领导、统揽全局、科学决策，为全市的发展明确了目标，指明了方向；市人大、市政协有效监督、大力支持、群策群力，为全市的发展凝聚了人心，增强了合力；全市广大干部群众团结一心、攻坚克难、奋发进取，为全市的发展作出了贡献，谱写了新篇。在此，我代表市人民政府，向付出辛勤劳动的全市广大干部群众，向各位人大代表、政协委员，向各民主党派、工商联、各人民团体、中直区直驻崇左各单位，向驻崇左人民解放军和武警部队官兵，向所有关心支持和帮助我市各项建设事业的各界人士，表示衷心的感谢和崇高的敬意 !

　　当前，崇左正处于转型发展的关键时期，经济社会发展中还存在不少困难和问题，主要表现在：经济总量依然偏小，稳增长压力较大；工业经济形势严峻，支柱产业效益下降；投资增长压力加大，项目建设推进不理想；财税收入质量不高，非税占比居高不下，可用财力偏少；农村基础设施落后，贫困面较大，城乡发展一体化任务艰巨；政府职能转变还不够， “ 四风 ” 问题还需深入整治等。对于这些困难和问题，我们将以对人民高度负责的态度，以勇于担当的精神，积极采取有效措施，努力克服和解决，决不辜负全市各族人民的期望 !

　　二、 2014 年目标任务和主要工作部署

　　今年是全面贯彻落实党的十八届三中全会精神、全面深化改革的开局之年，是全面完成 “ 十二五 ” 规划的关键一年。当前，国内外经济形势依然错综复杂，经济发展面临诸多困难和挑战，我市正处在爬坡过坎、不进则退的关键期，各项改革发展任务十分艰巨。但国内经济长期向好的趋势没有改变，我市面向东盟的区位优势进一步凸显，丰富独特的资源优势进一步发挥，国家加快沿边地区开发开放和崇左全境纳入北部湾经济区的政策 “ 红利 ” 进一步释放。特别是中国 — 东盟合作进入新的 “ 钻石十年 ” ，广西加快构建西南、中南地区开放发展新的战略支点，中央、自治区对崇左发展的期待更高、支持更大，全市人民求发展、盼富裕、奔小康的愿望更加强烈。我们一定要把握 “ 得天独厚 ” 的区位优势，抢抓 “ 千载难逢 ” 的重大机遇，树立 “ 机不可失 ” 的责任意识，乘势而上，实干争先，全面深化改革，奋力开创全市经济社会发展的崭新局面 !

　　 2014 年政府工作的总体要求是：全面贯彻落实党的十八届三中全会、中央经济工作会议、自治区党委十届四次全会、全区经济暨城镇化工作会议以及市委三届四次全会精神，围绕 “ 建设成为面向东盟开放合作的新高地、成为提升广西沿边开放水平的桥头堡 ” 的奋斗目标，坚持 “ 稳中求进 ” 的总基调，以 “ 加力开放合作、加快南崇经济带建设 ” 为主题，以开展党的群众路线教育实践活动为动力，把改革创新扩大开放贯穿于经济社会发展的各个领域各个环节，抢抓机遇，加快转方式、调结构、促转型，切实提高经济发展的质量和效益，更加注重改善民生，更加注重生态建设和环境保护，努力实现全市经济持续健康较快发展、社会和谐稳定，为与全国全区同步全面建成小康社会打下良好基础。

　　 2014 年政府工作的主要预期目标是：地区生产总值增长 10% ，财政收入增长 9% ，规模以上工业增加值增长 16% ，固定资产投资增长 18% ，社会消费品零售总额增长 13% ，外贸进出口总额增长 15% ，城镇居民人均可支配收入增长 10% ，农民人均纯收入增长 11% ，居民消费价格指数涨幅 4% 以内，万元生产总值能耗下降 1.76% ，完成自治区下达的减排任务，九年义务教育巩固率 79% ，高中阶段教育毛入学率 77% ，每万人口发明专利拥有量增长 50% ，城镇化率 36% ，城镇登记失业率控制在 4.2% 以内，城镇新增就业人数 1.8 万人，基本社会保险覆盖率 90% 。

　　为实现上述目标，我们将重点抓好以下八个方面的工作：

　　 ( 一 ) 全面深化改革，进一步增强发展新动力。

　　坚持从实际出发、从难题入手，主动作为，敢于突破，坚决破除影响发展的体制机制障碍，争创发展新优势、增强发展新动力。今年重点推进以下方面改革：

　　深化行政审批制度改革。加大取消和下放审批事项力度，进一步精简审批事项。对保留的审批事项优化办事流程，减少申办材料，简化办事环节，压缩办事时限。加强各级政务服务中心建设，加快电子政务建设。创新审批制度和审批方式，推行网上审批、并联审批和服务质量公开承诺等做法。加强行政审批领域防治腐败工作，推行 “ 阳光审批 ” 。

　　深化开放合作体制机制改革。推进北部湾经济区基础设施和公共服务共建共享机制建设，加快与北部湾其他五市之间通信、金融、旅游、产业、社会保障等领域的同城化进程。积极构建对接东盟的物流体系，全面落实中越客货运输协定，推进中越国际客货运输线路便利化。扩大口岸开放范围，推进互市贸易改革试点工作，在浦寨边贸区建设边民互市贸易中心。加快推动口岸经济区建设，扩大管辖范围，提升边境口岸经济区管委会服务能力。探索建立利用东盟劳动力资源协调管理机制。

　　深化农村改革。坚持家庭联产承包责任制和农民承包土地经营权不变，开展农村土地承包经营权确权试点工作，积极稳妥有序推进以 “ 小块并大块 ” 为重点的土地集中流转。允许社会民间资本参与土地整治，在农民自愿基础上，推动土地向农业龙头企业、专业大户、家庭农场或农民专业合作社流转。按国家政策赋予农民对承包土地占有、使用、收益、流转及承包经营权抵押、担保权能，允许农民以承包经营权入股发展农业产业化经营。深入推进征地制度改革，完善对被征地农民合理、规范、多元保障机制，并与规范农村集体经营性建设用地流转相协调。加快农村集体土地和宅基地确权登记发证工作，依法实行农村集体经营性建设用地和国有土地同等入市、同权同价。让农民在改革中获得更多实惠 !

　　深化财税金融体制改革。进一步完善预算绩效评价体系，今年市县两级公开财政、 “ 三公 ” 经费和部门预决算。稳步推进政府采购监督，完善政府购买服务工作，规范政府购买服务行为。推进沿边金融综合改革试验区建设，探索实施凭祥越南盾现金使用试点。推进面向东盟的大宗商品现货交易中心和跨境电商的金融服务平台建设。推动设立崇左国际商品交易所。加快信用担保体系建设，缓解中小企业融资难问题。

　　深化华侨农林场体制改革。按照 “ 体制融入地方、管理融入社会、经济融入市场 ” 的要求，以顺体制、活机制、强产业、惠民生为重点，统筹推进华侨农林场各项改革。发挥渠黎华侨林场和新和华侨农场示范带动作用，加快全市华侨农林场产业发展，增强 “ 造血 ” 功能。

　　进一步完善相关体制机制改革。在实施中进一步完善市与江州区国土资源管理、城市规划、城市建设与管理事权划分的事项，逐步形成灵活高效、运转协调、共同发展的城市管理体制。继续完善城市工业区管理体制改革，充分授予园区项目审批、规划管理、国土资源监管、财政收支等主要经济管理权限和相关行政管理职能，实现办理行政审批事项不出园区。创新发展模式，整合各投融资平台资源，重点发展壮大市城投公司、建卓公司等融资平台，启动发行城投企业债券。积极引入中小银行入驻我市，推动设立村镇银行，提高金融服务的密集度。

　　 ( 二 ) 加力开放合作，不断提升开放型经济水平。

　　继续围绕 “ 两加 ” 、 “ 两成 ” 目标，以东盟和粤港澳为重点，加力开放合作，推动沿边开发开放迈上新台阶。

　　推进南宁 — 崇左 — 凭祥对外开放经济带建设。主动融入广西建设 “ 新的战略支点 ” 大格局，用足用好用活国家加快沿边地区开发开放政策，加快推进南宁 — 崇左 — 凭祥对外开放经济带建设，积极搭建开发开放新平台。

　　推进凭祥重点开发开放试验区建设。尽快修编完善试验区实施方案，着手编制试验区发展规划、控制性详细规划等。推动试验区申报工作，争取尽快获得批准并启动建设。

　　推进跨境经济合作区建设。充分发挥凭祥综合保税区的区位作用。加强与国家有关部委和越南方面沟通协调，尽早完成中越凭祥 — 同登跨境经济合作区总体方案，力争上半年获得两国政府批准。积极争取上级支持，先行启动围网区一期规划和建设。

　　继续深化区域合作。主动融入国家 “ 海上丝绸之路 ” 、自治区 “ 双核驱动 ” 战略，全面参与北部湾经济区开放开发，主动对接粤港澳、桂台经贸合作等区域合作。争取与老挝他曲市、缅甸勃固市建立国际友城，拓展与欧美国家的友城关系。加强与南北钦防玉百六市各领域合作，促进区域经贸协调发展。

　　大力发展边境贸易。进一步完善边境口岸、互市贸易点的基础设施建设，争取在叫隘、那花、布局等边民互市点开展边境小额贸易。巩固传统贸易市场和产品，逐步开拓欧美、港澳台市场，继续做好纺织品、机电产品等传统大宗商品出口，进口适合与我市产业对接的资源性产品等，持续扩大外贸进出口规模。加快发展红木、农副产品、中草药等边贸加工业，促进边境贸易转型升级。加快崇左 ( 东盟 ) 国际商贸物流园、中国 — 东盟 ( 凭祥 ) 汽摩配专业市场等项目建设，推动商贸物流业快速发展。

　　推进互联互通建设。加快推进口岸升格和扩大开放工作。加快友谊关口岸 “ 一站式 ” 检查模式以及宁明爱店口岸验货场改造工程、凭祥市边境贸易货物监管中心等重点项目建设，提升口岸通关便利化。加快推进隆安至硕龙高速公路、湘桂铁路南宁至凭祥段扩能改造项目前期工作，力争开工建设崇左至水口高速公路；加快崇左至靖西高速公路、南宁新江经吴圩至扶绥一级公路建设，加快苏圩至凭祥、德保至天等、驮卢至大新等二级公路建设。继续推进左江崇左至南宁Ⅲ级航道工程、将军岭作业区建设，争取开工建设濑湍作业区项目，完成新环作业区项目前期工作。积极配合推进凭祥 — 河内高速公路、南宁 — 凭祥 — 同登 — 河内铁路、水口 — 驮隆中越界河公路二桥项目建设。

　　进一步强化招商引资。建立全市招商引资项目库并进行动态管理，进一步创新招商方式，大招商、招大商，力争在引进国内外知名企业上有新突破，确保今年引进市外境内项目资金 400 亿元以上，全口径实际利用外资 2 亿美元以上。

　　 ( 三 ) 着力稳增长、转方式、调结构，不断扩量提质增效。

　　坚持量与质同步，在扩量中提质，在提质中转方式、调结构，促增长，进一步增强经济发展的质量和效益。

　　大力推进结构调整。深入发展糖业循环经济，改造提升生态型锰精深加工产业，推进产品高端化、精品化、差异化发展。着力培育发展铜、稀土、铝等产业，优化产业结构。培育发展边贸加工产业、特色食品加工业，变过境贸易为加工贸易。培育和打造品牌，加强特色品牌保护和利用。

　　培育引进一批大型企业。加快实施企业技改兼并重组行动计划，支持现有企业进行整合和重组，培育发展一批主业突出、品牌知名度高、国际竞争力强、辐射带动作用大的优势企业。到今年底，力争全市拥有主营业务收入超亿元企业 82 家以上，超 10 亿元企业 15 家以上，超 30 亿元企业在现有基础上有一定的增长。

　　加快推进项目建设。继续落实市领导联系重大项目责任制，加大服务力度，积极协调解决问题，加快项目建设步伐，重点抓好市层面统筹推进的 201 项重大项目建设。优先保障重点产业、民生工程和重大基础设施项目用地需求。完善联合审批制度，创造条件加快推动项目前期工作。

　　加强节能减排工作。建立健全节能减排监测预警体系。扎实推进工业、建筑、交通运输、公共机构等重点领域节能。完善环境监测、监察和应急体系，着力抓好主要污染物减排。

　　巩固和发展非公有制经济。毫不动摇鼓励、支持、引导非公有制经济发展，加大政策扶持和支持力度，提高产业发展资金对非公经济的扶持比重。力争启动建设中小企业投资区。继续开展 “ 千家企业大走访 ” 活动，积极为企业排忧解难。重视非公有制企业家队伍建设，调动企业家积极性、创造性。积极承办全国家居业知名企业崇左行暨民营企业家联谊会，以会促商，争取引进一批知名家具企业落户崇左。

　　 ( 四 ) 夯实产业发展基础，不断壮大综合经济实力。

　　发挥南崇经济带 “ 陆路东盟黄金通道 ” 作用，引导产业、项目、企业、园区向南崇经济带集聚发展，提高产业聚集度。

　　延伸拓展产业链。巩固发展支柱特色产业，积极培育新兴产业，推动产业转型升级，提升产业档次和层次。糖业方面，大力发展蔗糖循环经济及深加工项目，重点推进崇左东亚、湘桂糖业、中粮屯河等蔗糖循环经济项目建设。推进金冠等糖果加工项目，力争年内竣工投产。锰业方面，重点推进中信大锰布东锰生态工业园锰系新材料深加工及配套等项目开工建设，确保沙钢锰业硅锰合金技改扩建项目一期、新振锰业电解金属锰项目一期竣工投产，二期项目启动。有色金属及建材业方面，加快南国铜业项目建设，力争在 2015 年底前竣工投产、发挥效益。加快中铝稀土产业园建设，启动稀土分离生产线二期项目建设。加快崇左低品位铝土矿综合开发利用项目前期工作，力争早日开工建设。推动南方、红狮水泥项目竣工投产。红木产业方面，力争红木加工基地首批入园企业上半年全部竣工投产，二期 25 家企业全部开工建设，争取年内实现规模以上红木加工业产值 10 亿元。新兴产业方面，抓好宁明东亚生物质能发电项目、宁明诺沃奇等新能源类项目建设，加快推动科创集团红木商贸城、健康城等项目前期工作。食品加工产业方面，重点发展坚果加工和糖果休闲食品产业。

　　推动园区建设提质升级。进一步完善园区规划，明确园区定位，持续优化区域产业布局，实现错位发展。积极争取自治区支持，集中财力、资源等要素推进崇左城市工业区、中国 — 东盟青年产业园建设，每个园区培育形成 1—2 个支柱产业，促进园区提质升级。尽快完成中泰 ( 崇左 ) 产业园控制性详细规划、产业发展布局规划，积极争取成为中泰两国政府合作推进的园区。广西 · 东盟特色食品产业园争取有 2 个项目建成投产。加快产城融合，重点推动崇左城市工业区与中心城区融合发展。力争全市工业园区总产值突破 400 亿元。

　　做大工业经济规模。深入实施 “ 工业强市 ” 战略，树立 “ 工业优先，项目带动 ” 的理念，围绕工业跨越发展 “1915 工程 ” 、 “ 十大行动计划 ” ，开展 “ 工业项目建设服务年 ” 活动，在项目审批、项目用地、优惠政策等方面优先向工业倾斜。积极发展实体经济和总部经济，支持企业总部或者研发中心、物流中心等落户崇左。加快推进一批重大工业项目开竣工，引领带动工业投资和工业发展。

　　提高能源保障能力。进一步加强电网建设，完善城市配网网架，提高农村电网供电能力。启动 500 千伏崇左二站输变电工程项目前期工作，推进 220 千伏聚龙、江南送变电工程、 110 千伏石林送变电工程等项目。推进锰电结合，鼓励企业建设自备电厂。加快崇左电厂项目前期工作。大力推进县县通天然气工程。

　　 ( 五 ) 加快发展现代农业，促进农业增效、农民增收。

　　切实保障粮食安全。落实强农惠农富农政策和粮食生产责任制，加强基本农田保护，积极发展超级稻、玉米、马铃薯等粮食作物，确保全市粮食播种面积稳定在 181 万亩以上、总产量 50 万吨以上。加强粮食仓储设施建设和维修改造，保障粮食储存能力。

　　做大做强特色效益农业。深入实施蔗糖产业 “4331” 工程，稳定甘蔗种植面积在 430 万亩以上，稳步提高甘蔗产量。加快甘蔗良种品种培育、引进、示范、推广。新增甘蔗高效节水灌溉工程 20.5 万亩，先期实施 20 万亩以上优质高产高糖甘蔗示范基地建设。实施 “ 菜篮子 ” 工程，大力推广农作物间套种技术，加大农业秋冬产业开发。培育壮大特色水果、木薯和中药材等特色农业，积极发展休闲生态观光农业。大力发展养殖业，稳定生猪生产，推进优势规模养殖和特色养殖。加快农产品流通体系建设，发展多种形式的产销衔接。推动开展农产品原产地认证工作。

　　加强水利基础设施建设。加快左江治旱工程项目前期工作，争取驮英水库和灌区项目获国家发改委批准立项。加快推进病险水库除险加固、中小河流治理、水毁工程修复等重大水利项目建设。积极争取第七批中央财政小型农田水利重点县项目，解决 13.7 万农村人口及中小学师生饮水不安全问题。

　　大力推进农业生产机械化。做好农机购置补贴工作。耕种收综合机械化水平达到 46.5% 。建设甘蔗生产机械化示范基地 30 万亩，着力解决甘蔗收获机械化难题，降低甘蔗种收成本。

　　加快构建新型农业经营体系。推进家庭经营、集体经营、合作经营、企业经营等共同发展的农业经营方式，发展适度规模经营。大力培育新型农业经营主体，年内新增农民专业合作社 80 个以上。积极引导推进土地流转，力争完成 “ 小块并大块 ” 土地整治 60 万亩以上。

　　 ( 六 ) 积极稳妥推进新型城镇化，打造南崇城镇带。

　　以 “ 东盟、生态、壮乡、现代 ” 为基本元素，启动新型城镇化建设规划编制，加快南崇城镇带建设。力争今年底全市新增城镇人口 5 万人以上。

　　加快中心城市建设。以项目建设为依托，重点推进 “ 一江一园一改一桥两校三中心八路 ” 建设，不断拉大中心城市框架。加快龙峡山西路延长线、城区污水管网二期工程等项目建设，争取尽快竣工投入使用；加快环城西路、崇左大桥、龙腾湖风情街等项目前期工作，争取开工建设。加快推进第七届广西园博会规划设计工作，全面开工建设基础设施项目。加大公共服务设施建设力度，完善城市功能，增强中心城市综合承载能力。至今年底，中心城市建成区面积要达到 30 平方公里，人口达到 17 万人。

　　推进大县城和小城镇建设。重点发展凭祥及扶绥，建立两个市级副中心城市，其他县城不断扩容提质、彰显特色；加快凭祥城乡一体化示范区建设，启动实施宁明 — 凭祥同城化发展，推进扶绥空港 — 南宁吴圩融合发展。积极培育特色城镇，因地制宜抓好旅游、边贸、产业等特色城镇规划和发展。继续推进名镇名村建设和城乡风貌改造等工程，每个县 ( 市、区 ) 抓好 1—2 个示范乡镇建设。

　　加快制定实施城镇化配套政策。进一步放宽户口入城迁移准入条件，全面建立城乡统一的户口登记管理制度。完善进城务工人员社会保障制度，探索建立城乡统一的就业制度，鼓励和吸引农民、外来经商置业人员、专业人才进城落户。拓展城镇化建设投融资渠道，鼓励和支持社会民间资本参与城市公用设施建设，共同建设和经营城镇。

　　 ( 七 ) 加快旅游产业跨越发展，打造边关旅游胜地。

　　充分挖掘崇左旅游的巨大潜力，发挥资源优势，加快推动旅游产业跨越发展。

　　狠抓旅游项目建设。大力实施旅游 “25671” 工程。重点抓好列入自治区 2013—2015 年重大旅游项目的 “ 大德天景区 ” 升级改造项目、龙州上金国际生态休闲度假旅游区综合开发项目等重大项目建设。加快旅游饭店、游客服务中心、旅游交通路网等基础设施建设。积极推进特色旅游名县创建工作。

　　培育文化旅游品牌。挖掘民族特色，加快文化与旅游融合发展。继续抓好花山岩画文化景观申遗工作，力争列入 2016 年中国申遗项目。培育打造红八军红色旅游和中越跨国红色旅游品牌。依托红木资源，推动我市红木产业向红木鉴赏、文化旅游拓展。积极推进德天瀑布景区、友谊关景区创 5A 和其他景区创 4A 工作。

　　推进旅游区域合作。充分发挥边关风情和中越边境旅游异地办证业务的优势，扩大发展边境旅游业。大力推进中越国际旅游合作区建设，推动中越两国政府尽快签订《中越德天 — 板约瀑布地区旅游资源保护和开发协定》；积极争取将凭祥浦寨和越南新清作为中越友谊关国际旅游合作区先行先试区加以推进，尽快开通中越边境 “ 一日游 ” 线路。加快推进免税购物区申请工作。进一步融入北部湾大旅游圈，推动旅游业区域联合发展。

　　加强生态文明建设。继续组织实施主体功能区规划，积极推进生态市建设。深入实施 “ 美丽广西 · 清洁乡村 ” 活动，抓好 “ 一江六线多节点 ” 村屯建设。抓好退耕还林工作。扎实推进 “ 国家森林城市 ” 创建工作，积极打造 “ 崇左市城区万亩珍贵树种种植示范基地 ” ，争取成功申报国家珍贵树种培育示范市、 “ 中国木棉之乡 ” 。我们一定要保持好山青水绿天蓝的生态宜居品牌，让生态 “ 红利 ” 惠泽子孙后代 !

　　 ( 八 ) 集中力量抓好民生事业，促进社会和谐发展。

　　大力发展教育事业。坚持优先发展教育，树立 “ 不建楼堂建学堂 ” 的理念，抢抓广西教育大发展的有利时机，积极创办优质教育，实施 “ 名校名师 ” 战略，从今年起到 2017 年，多渠道筹措各类资金建设和完善 100 所以上学校。加快学前教育公办幼儿园建设。优化学校布局调整，加快城乡义务教育学校标准化建设。完善城区教育基础设施，打造特色品牌学校，力争今年 9 月城南小学和崇左市机关幼儿园实现开学。推动普通高中优质多样特色发展。深化市、县职业教育综合改革，加快推进崇左国门职业教育园区建设，建好广西崇左东盟职业教育学院。

　　扎实推进扶贫开发。积极推进滇桂黔石漠化片区区域发展，开展贫困村整村推进扶贫开发攻坚大会战。整合资金 6 亿元，大力实施兴边富民行动大会战。继续推进 “ 领导挂点、单位包村、干部联户、第一书记和指导员驻村当代表 ” 的工作责任制；抓好社会扶贫，引导和鼓励民间组织、企业家、个体户等参与扶贫开发。力争全年减少贫困人口 6 万人以上，让贫困群众尽快富裕起来，共享美好幸福生活 !

　　稳定和扩大就业创业。实施更加积极的就业政策，开展职业技能大培训工程、就业援助及农民工返乡创业帮扶活动，不断扩大就业创业规模，保持就业局势稳定。力争新增农村劳动力转移就业 4.6 万人，实现累计在外稳定转移就业达 55 万人以上，实现劳务经济总收入 70 亿元以上。

　　健全完善社会保障体系。进一步扩大社会保障覆盖面，适度提高各项社会保险待遇水平。落实全国统一的城乡居民基本养老保险制度。加快推进社会保险 “ 金保 ” 工程建设，年内实现社会保险 “ 五险合一 ” 管理系统上线运行。推进幸福院和 “ 民政项目园 ” 建设，加强社会救助、城乡低保、五保供养、救灾等工作。加强养老、残疾人服务产业建设。完成自治区下达的农村危房、保障性安居工程、棚户区改造建设任务。

　　强化人才科技工作。扎实推进人才强市战略，继续面向全国公开引进各类高层次人才，加强人才小高地建设。培育农村专业化人才。推进创新驱动发展，积极创建高新技术企业孵化器，加快崇左市科技创业园建设。

　　加快发展医疗卫生事业。深入推进医改工作，巩固完善基本药物制度。巩固新农合制度，确保参合率达到 95% 以上。完善新农合 “ 一卡通 ” ，推进城乡居民大病医保试点工作，推进乡村卫生服务一体化管理。加强基本公共卫生服务工作，有效防控艾滋病等重大疾病。开工建设市人民医院内科综合楼、市儿童医院、市妇幼保健院等项目。落实国家、自治区计划生育新政策，全面推进诚信计生。进一步加强出生人口性别比综合治理。深入实施免费孕前优生健康检查项目，提高出生人口素质。

　　繁荣发展文化体育事业。加快市文化艺术中心建设，力争上半年向市民开放。继续抓好村级公共服务中心建设和 “ 百团千场 ” 群众文化活动。继续实施公共体育体系建设，广泛开展全民健身活动。加快推进市体育中心建设。加快广播影视发展，开播一个电台新频率。

　　积极扩大消费需求。继续加大商品市场建设力度，努力构建以崇左市区为中心、以县城为次中心的区域性商贸中心。加快农村市场体系建设。积极推动假日消费和展会消费。加强市场价格宏观调控，保持我市物价总水平基本稳定。

　　继续实施为民办实事工程。筹措资金 31.4 亿元，实施社保、健康、教育、强基、安居、土地整治、农补、生态、文化、交通等 10 项为民办实事工程。

　　加强社会治理。改进社会治理方式，加强社区管理服务创新，提高社会治理水平，进一步提升全市公众安全感和满意度。继续深化平安崇左建设，强化社会治安综合治理，进一步加大对各类违法犯罪的专项打击力度。加强边境管控，推行边境社会管理信息化。深入开展社会矛盾排查化解工作，健全矛盾化解机制。改革信访工作，畅通民意表达渠道，落实及时就地解决群众合理诉求的措施。全面推行重大决策社会稳定风险评估机制，加强突发事件应急处理工作。进一步加强防震减灾工作。深入开展安全生产隐患大检查，坚决遏制各类重大事故发生。全力推进食品药品安全综合整治，抓好农产品质量和食品安全监管。

　　巩固民族团结。继续深入开展民族团结进步创建活动，抓好民族特色村寨建设。加强宗教工作，创和谐寺观教堂，促进民族团结、宗教和谐。

　　加强国防建设。加强国防动员、国防后备力量建设和人民防空工作。积极开展全国双拥模范城创建活动。

　　三、努力建设法治型政府、服务型政府

　　做好今年政府工作，必须着力加强政府自身建设，以转变政府职能，加强经济运行调节、法治建设和作风建设为重点，不断提高政府的公信力、执行力、服务力和创新力，切实履行党和人民赋予的神圣职责，努力打造人民满意的法治型、服务型政府。

　　 ( 一 ) 加快政府职能转变。严格管理机构编制，推进市县政府机构改革，优化政府机构设置和职能配置。推进简政放权，进一步理顺体制机制，加强平台建设和管理，使资源更多地依据市场进行有效配置。严格绩效管理，确保责任落实、责权一致。推进事业单位分类改革和社会组织管理改革。

　　 ( 二 ) 加强经济运行调节。强化对宏观经济形势的研判，把握宏观经济运行周期和规律，在产业布局、经济运行过程中适时适度进行反周期操作，增强产业抗风险能力。统筹协调和服务工业企业的产运销，通过争取用电支持和使用价格基金，促进锰业扩大生产。加强政府性债务管理，防范债务风险。强化部门的预算执行主体责任，切实加快支出进度。做好全面建成小康社会的统计监测分析和第三次经济普查工作。

　　 ( 三 ) 加强法治政府建设。坚持开门纳谏、广开言路，推进决策科学化、民主化与制度化。强化重大决策跟踪反馈和责任追究。加强政务公开，推进政社分开、政事分开和行政权力透明运行。健全行政复议案件审理机制，纠正违法或不当行政行为。坚持依法行政，深入开展法治城市、法治县 ( 市、区 ) 、法治村 ( 社区 ) 创建活动。自觉接受人大及其常委会监督和人民政协民主监督，认真听取各民主党派、工商联、无党派人士、人民团体和社会各界人士的意见。充分发挥行政监察、审计监督等专门机构的作用，高度重视人民群众监督和新闻舆论监督。

　　 ( 四 ) 加强作风建设。深入开展党的群众路线教育实践活动，认真执行中央 “ 八项规定 ” ，坚决反对和纠正 “ 四风 ” ，深入基层问政、问需、问计，密切与人民群众的联系，树立为民务实清廉新形象。加强政风行风建设、深化民主评议活动，深入实施问政工程。围绕人民群众关心的热点问题，继续开展好 “ 三服务 ” 、 “ 四清理 ” 活动，维护人民群众切身利益。严肃查处违纪违法案件，纠正损害群众利益的不正之风。深入推进惩治和预防腐败体系建设，健全权力运行制约和监督体系的相关制度。树立新的政绩观，完善考评机制和激励办法，更加注重把民生改善、科技创新、社会进步、生态效益、政府债务管理等指标和实绩作为考核干部政绩的重要依据。

　　各位代表，形势催人奋进，发展任重道远。让我们紧密团结在以习近平同志为总书记的党中央周围，在市委的坚强领导下，解放思想、锐意进取、扎实工作，全面深化改革，奋力完成今年各项目标任务，为与全国全区同步建成小康社会，把崇左建设成为面向东盟开放合作的新高地、成为提升广西沿边开放水平的桥头堡而努力奋斗 !
